# Gastrointestinal helminths may affect host susceptibility to anthrax through seasonal immune trade-offs

**DOI:** 10.1186/s12898-014-0027-3

**Published:** 2014-11-12

**Authors:** Carrie A Cizauskas, Wendy C Turner, Bettina Wagner, Martina Küsters, Russell E Vance, Wayne M Getz

**Affiliations:** Department of Environmental Science, Policy, and Management, University of California, Berkeley, Berkeley, CA USA; Centre for Ecological and Evolutionary Synthesis, Department of Biosciences, University of Oslo, Oslo, Norway; Department of Population Medicine and Diagnostic Sciences, College of Veterinary Medicine, Cornell University, Ithaca, NY USA; Ministry of Environment and Tourism, Windhoek, Namibia; Department of Molecular and Cell Biology, University of California, Berkeley, Berkeley, CA USA; School of Mathematical Sciences, University of KwaZulu-Natal, Durban, South Africa; Department of Ecology and Evolutionary Biology, Princeton University, Princeton, NJ USA

**Keywords:** Bacteria, Ecological immunology, Endoparasites, Host-parasite interactions, Microparasites, Coinfections immunological trade-offs, Disease ecology, Seasonality

## Abstract

**Background:**

Most vertebrates experience coinfections, and many pathogen-pathogen interactions occur indirectly through the host immune system. These interactions are particularly strong in mixed micro-macroparasite infections because of immunomodulatory effects of helminth parasites. While these trade-offs have been examined extensively in laboratory animals, few studies have examined them in natural systems. Additionally, many wildlife pathogens fluctuate seasonally, at least partly due to seasonal host immune changes. We therefore examined seasonality of immune resource allocation, pathogen abundance and exposure, and interactions between infections and immunity in plains zebra (*Equus quagga*) in Etosha National Park (ENP), Namibia, a system with strongly seasonal patterns of gastrointestinal (GI) helminth infection intensity and concurrent anthrax outbreaks. Both pathogens are environmentally transmitted, and helminth seasonality is driven by environmental pressures on free living life stages. The reasons behind anthrax seasonality are currently not understood, though anthrax is less likely directly driven by environmental factors.

**Results:**

We measured a complex, interacting set of variables and found evidence that GI helminth infection intensities, eosinophil counts, IgE and IgGb antibody titers, and possibly IL-4 cytokine signaling were increased in wetter seasons, and that ectoparasite infestations and possibly IFN-γ cytokine signaling were increased in drier seasons. Monocyte counts and anti-anthrax antibody titers were negatively associated with wet season eosinophilia, and monocytes were negatively correlated with IgGb and IgE titers. Taken together, this supports the hypothesis that ENP wet seasons are characterized by immune resource allocation toward Th-2 type responses, while Th1-type immunity may prevail in drier seasons, and that hosts may experience Th1-Th2 trade-offs. We found evidence that this Th2-type resource allocation is likely driven by GI parasite infections, and that these trade-offs may render hosts less capable of concurrently mounting effective Th1-type immune responses against anthrax.

**Conclusions:**

This study is one of the first to examine laboratory-demonstrated Th1-Th2 trade-offs in a natural system. It provides evidence that seasonally bound pathogens may affect, through immunology, transmission dynamics of pathogens that might otherwise not be seasonally distributed. It suggests that, by manipulating the internal host ecosystem, GI parasites may influence the external ecosystem by affecting the dynamics of another environmentally transmitted pathogen.

**Electronic supplementary material:**

The online version of this article (doi:10.1186/s12898-014-0027-3) contains supplementary material, which is available to authorized users.

## Background

Microparasites (bacteria, viruses, fungi, protozoa) and macroparasites (helminths, arthropods) are important components of both external and within-host ecosystems [1]. The majority of animal and human hosts are co-infected with pathogens [2], often a mix of micro- and macroparasites that can interact with each other to modify pathogen transmission, virulence, and host availability. While pathogens can compete with each other for within-host niches [3], the majority of interactions appear to occur indirectly via the immune system [4,5]. Through immunomodulation, infections can increase host susceptibility to other parasites [6,7], enhance the intensity of other pathogen infections [5,8], increase disease severity and pathology [6,9], and increase disease duration [5,10].

Immunomodulation and immune tradeoffs are particularly strong in mixed microparasite-macroparasite infections. Intracellular pathogens (microparasites) generally cause the mammalian adaptive immune system to mobilize T-helper-1 (Th1) cells, while extracellular pathogens (macroparasites) usually trigger T-helper-2 (Th2) cells. The pathways leading to and from Th1-type responses and Th2-type responses are mutually cross-regulated; thus, hosts have difficulty simultaneously mounting effective Th1 and Th2 responses [11,12]. Many studies have demonstrated that helminth infections are particularly adept at skewing immune responses toward the Th2 arm [11,13] and downregulating Th1 immunity even in the face of microparasitic coinfections [6,9,14-16]. Chronic helminth infections may also cause host immunosuppression, resulting in maintenance of the immunomodulating worm infection [17] and increased susceptibility to coinfections [18].

While many studies regarding the effects of coinfections and immune trade-offs have been conducted in laboratory settings, similar studies in wildlife are rare. Fewer studies have been conducted regarding disease and immune seasonality, as these are challenging to model in laboratory settings and require difficult, longitudinal samplings in natural systems. Seasonality is especially prominent in directly transmitted helminth infections, as free-living stages require temperature and moisture thresholds to develop and survive outside of hosts [19,20]. Given the seasonality of these infections and their propensity to immunomodulate hosts, it is likely that helminths can help drive seasonal patterns of coinfections, but this hypothesis has rarely been examined. Here we present what we believe to be the first longitudinal study simultaneously examining coinfections, seasonality, and complex immunity in wildlife hosts.

Over three years, we examined a natural population of plains zebra (*Equus quagga*) in Etosha National Park (ENP), Namibia. We examined coinfections with ectoparasites (ticks), gastrointestinal (GI) helminths, and anthrax in concert with several measures of immune function in these hosts. These three parasites are the only known pathogens infecting zebra hosts in ENP; rabies does exist in this system, but there is no evidence that zebra play a role in its dynamics, and there is as yet no evidence of tick-borne pathogens in these ENP hosts Etosha Ecological Institute, EEI, unpublished data; [21]. While ENP zebra have a nearly 100% prevalence year-round with GI helminths, they experience a significant increase in infection intensity in the wet season compared to in the dry [22]. These new helminth infections are constrained to the wet season; the eggs and larvae require for survival moisture and moderate temperatures over the 1–2 week period of development in the external environment before they become infectious to new hosts [23,24]. The wet season is also when ENP plains ungulates experience annual outbreaks of anthrax, a bacterial infection caused by the ingestion of *Bacillus anthracis* spores from the environment [25,26]; zebra account for 52% of all anthrax cases in ENP, and 57% of all anthrax cases occur in March and April at the middle and end of the rainy season [27]. The reasons for anthrax seasonality, however, are not yet understood. The traditional spore concentration hypothesis (i.e. rains wash heavy *B. anthracis* spores into depressions, leaving large spore concentrations in small areas when puddles dry and accounting for dry season anthrax outbreaks in several systems) [28] cannot account for the ENP rainy season outbreaks. There is as yet no definitive evidence that anthrax can multiply in the soil in natural systems [25]; though see [29,30], and thus anthrax spore levels likely do not increase under seasonal conditions. Given the endemic nature of anthrax in ENP, the prolonged survival times of spores in the environment [28,31], the fact that anthrax deaths and sublethal infections do occur throughout the year in this system [32], it is likely that animals come into contact with anthrax spores in all seasons, though some hosts may also ingest more *B. anthracis* in soil during wetter times [27]. While multiple factors are likely involved in the timing of anthrax outbreaks, we hypothesize that seasonal changes in host coinfection and immune factors may influence host susceptibility to this environmental pathogen. Anthrax can cause death within hours to days [33], though there is evidence that even very susceptible host species can experience a sublethal dose of anthrax and survive, in part, due to a humoral immune response against the anthrax protective antigen (PA) toxin [32,34]. These anti-PA antibodies have been shown to be essential for adaptive protection against anthrax and can mature through memory responses with multiple infections, though otherwise tend to last less than six months [32,35,36]. As a bacterial infection, anthrax provokes a primarily Th1-type immune response, a response in opposition to the Th2-type immune response driven by helminth infections [37]. Thus, we hypothesize that the seasonally constrained increase in helminth infections in ENP zebras result in immunomodulation that increases host susceptibility to anthrax, even in the face of likely increased immune resources during a time of nutritional surplus [38].

We address this possibility by i) examining the host immune responses and immunomodulatory relationships that correlate with each of the three pathogens being examined, and ii) determining if GI parasite infection intensities most strongly correlate with host Th2-type immune resource allocation that may influence immunity to anthrax.

## Results

### Seasonal comparisons

Our study design involved the sampling of zebra over five seasons (two wet, three dry), resampling the same individuals as many times as possible over those five events to control for individual variation. We compared several immune and pathogen measures between seasons. To control for nutritional status and resource allocation, we measured total white blood cell (WBC) counts and hematocrits (HCT). Total WBC counts are a broad indicator of how much a host has invested in immune resources [39]. Hematocrit is a measure of percent of red blood cells per unit of blood, with a higher hematocrit often reflecting a higher nutritional state [40]. Eosinophils are white blood cells often important for eliminating helminths, are quite specific for macroparasite infections, and have been shown to be a good measure of Th2 responsiveness [11,41-43]. Monocytes are white blood cells that are recruited and activated by interferon-gamma (IFN-γ) in Th1 immune reactions, and were used as a measurement of potential Th1 immune activity [11]. Immunoglobulin E (IgE) is an antibody isotype mediated by the Th2-associated cytokine interleukin 4 (IL-4) and important, and specific, in fighting against helminths [11]. Immunoglobulin G, subtype b (IgGb) is the most prevalent antibody isotype in equine serum [44]. While IgGb is important in the protective response against intracellular pathogens, suggesting a Th1-associated response, Hooper-McGrevy *et al.* [45] found that IgGb is likely a Th2-related antibody in horses. Antibodies against the protective antigen (PA) toxin component of anthrax have been shown to be essential for adaptive protection against anthrax [35,36,46]. As a bacterial infection, anthrax provokes primarily a Th1 type immune response [37,47]. IFN-γ is a key Th1 cytokine that activates macrophages early in infection. IL-4 is an important Th2 cytokine that induces antibody isotype switching to IgE and augments recruitment of eosinophils in response to the presence of helminths [11,48].

**Figure 1:**
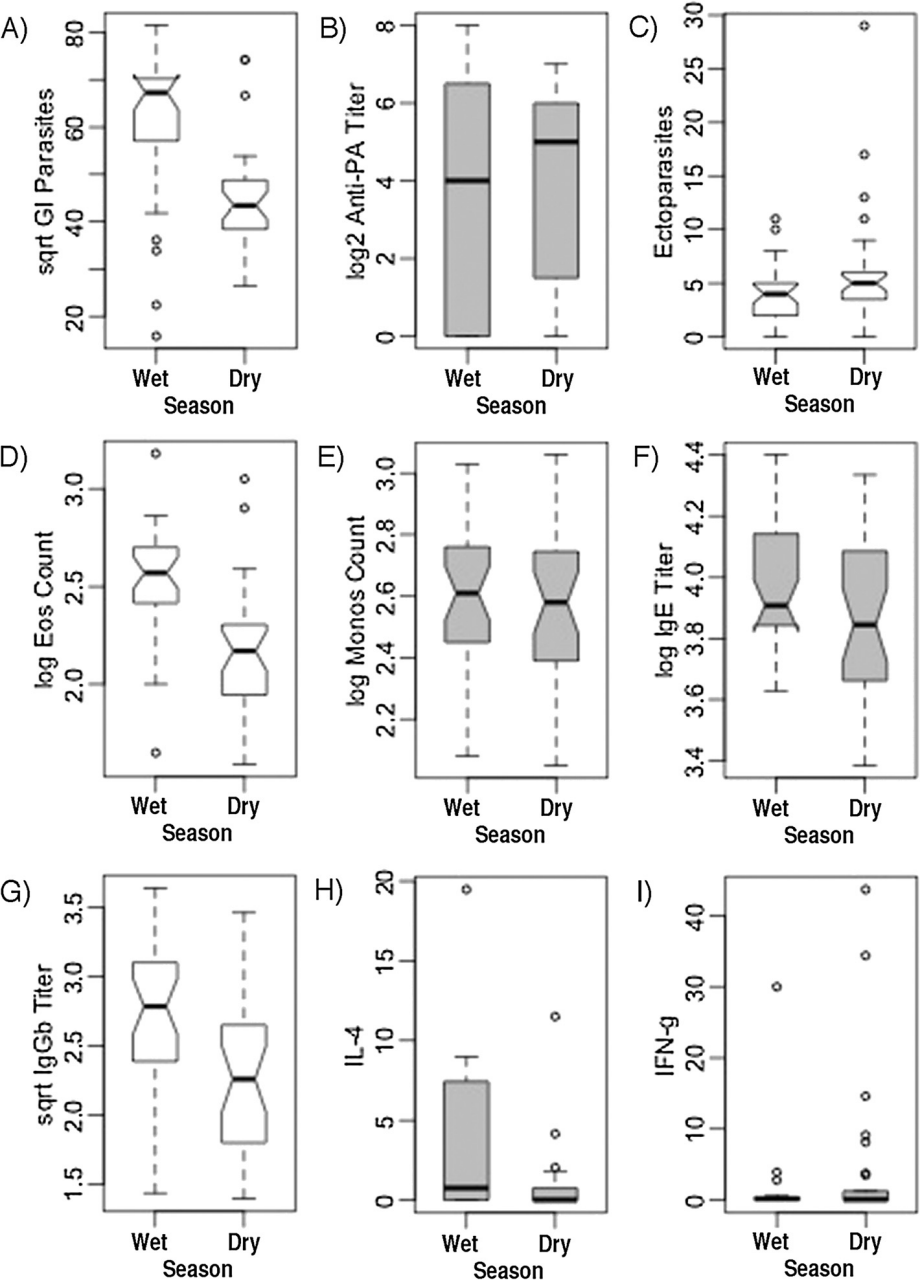
**Pairwise comparisons of pathogens and immune factors between seasons.** Comparisons are for the same individuals resampled twice (individual-level comparisions). Panels are labeled for ease of reference within the text. Boxplots in white are for variables that are significantly different from each other by *t* tests or Wilcoxon signed rank tests, whereas grey boxplots are not significantly different.

We first compared data from only the first sampling events for animals between the two season types. This ensured that we were only comparing unique individuals, and their population-level effects, between seasons. We then did pairwise comparisons across the same animals, between seasons, for first and second captures only to examine individual level effects; this allowed us to account for individual-level variation that otherwise might have obscured patterns at the population level. We found highly statistically significant differences between seasons for GI parasites and eosinophil counts for both individual and population-level comparisons (Figure 1A and D; Table 1). IgGb titers (Figure 1G) were nearly significantly different between seasons at a population level, and were significantly different between seasons for individual-level comparisons; thus, our paired comparisons aided in elucidating patterns otherwise obscured by individual variation. Similarly, ectoparasite counts were only significantly different between rain groups for individual-level comparisons (Figure 1B, E, and F; Table 1). In addition, we found that both WBC counts and HCT were significantly higher in the wet season for both population-level (*p* = 0.007, *p* = 0.003, respectively), and for individual-level comparisons (*p* = 0.001, *p* = 0.020, respectively).

**Table 1:** **Two-tailed welch's** ***t*** **tests and wilcoxon rank sum tests comparing pathogen and immune variables between seasons**

**Variable**	***N*** **(Wet, Dry)**	**df**	***t*** **,** ***U*** **, or** ***T***	***p*** **-value before correction**	**Holm's corrected** ***p*** **-value**	**Mean Difference** ^**#**^	**Higher Group**
Population-Level							
GIsqrt	45, 24	66.9	5.36	0.000***	0.000***	1771	Wet
log2PA	45, 24		643^+^	0.095.	0.379	4.34	Wet
Ecto	45, 24		493^+^	0.553	1.000	0.15	Dry
logEos	45, 24	55.3	5.07	0.000***	0.000***	240	Wet
logMonos	45, 24	43.1	0.45	0.653	1.000	34.1	Dry
logIgE	45, 24	32.6	0.40	0.690	1.000	1.01	Dry
IgGsqrt	45, 24	59.1	2.36	0.022*	0.109	1.44	Wet
IL-4	5, 37		101^+^	0.686	1.000	15.2-fold	Wet
IFN-γ	6, 37		78.0^+^	0.249	1.000	3.0-fold	Dry
Individual-Level							
GIsqrt	32, 32	31	5.35	0.000***	0.000***	1877	Wet
log2PA	32, 32		273^+^	0.663	1.000	0.19	Dry
Ecto	32, 32		78.0^+^	0.005**	0.022*	2.41	Dry
logEos	32, 32	31	5.11	0.000***	0.000***	214	Wet
logMonos	32, 32	31	0.19	0.850	1.000	17.3	Dry
logIgE	32, 32	31	1.99	0.056.	0.167	1.82	Wet
IgGsqrt	32, 32	31	2.70	0.011*	0.044*	1.85	Wet
IL-4	9, 9		8.50^+^	0.608	1.000	1.6-fold	Wet
IFN-γ	6, 6		9.50^+^	0.916	1.000	1.1-fold	Dry

Note.— “Population-Level” = first animal samplings compared between seasons; “Individual-Level” = first and second samplings of the same individuals compared between seasons.

^+^are Wilcoxon rank sum test results, using the test statistic *U* for unique comparisons, or are Wilcoxon signed rank test results, using the test statistic *T* for paired comparisons.

^#^Mean differences are differences between non-transformed means in the two seasons; fold differences are shown for IL-4 and IFN-γ, as per convention. Units for mean differences are epg for GIP; log_2_ titer for log_2_PA; number of ticks for Ecto; cells/μl of blood for Eos and Monos; μg/ml for IgE; and mg/ml for IgG.

. *p* ≤ 0.1; *p ≤ 0.05; **p ≤ 0.01; ***p ≤ 0.001.

We found no statistically significant differences when comparing IL-4 transcript ratios between seasons for all animals (Wilcoxon *U* = 232, *N*_1_ = 17, *N*_2_ = 36, *p* = 0.145; 4.6-fold increase in wet season), or for first or paired captures (Figure 1H; Table 1). However, we found a mean 15.2-fold increase in the mean transcript in the wet season in our population-level analysis. We found no statistically significant differences when comparing IFN-γ transcript ratios between seasons for all animals (Wilcoxon *U* = 245, *N*_1_ = 16, *N*_2_ = 37, *p* = 0.317; 2.4-fold increase in dry season), or for first or paired captures (Figure 1I; Table 1). Comparing mean ratios in the different seasons, however, we found a 3.0-fold increase in transcript in the dry season in our population-level analysis. Thus, we conclude that there is some evidence showing an increase in IL-4 production during wetter times, and for an increase in IFN-γ production during drier times but more data are needed.

### Parasite coinfection models

In the best fitting GI parasite model, increased cumulative rainfall two months prior to sampling (hereafter simply "rainfall") significantly predicted higher GI parasite loads, as expected from seasonal analyses (Figure 1A). However, higher eosinophil counts significantly predicted decreased GI parasite infection intensity (Table 2). Higher IgE titer nearly significantly (*p* = 0.085) predicted decreased GI parasite loads. While anti-PA titer was not statistically significant in the model, its negative coefficient suggests that higher magnitude anti-PA titers may be associated with lower GI parasite counts (Table 2).

**Table 2:** **Maximum likelihood estimates for the best fit generalized estimating equation pathogen and immunity models**

**Response**	**Coefficients**	**Estimate ± SE**	**Wald statistic**	***p*** **-value**	**ΔQIC from full model**
GIsqrt	Intercept	3.83 ± 0.06	4614	0.000***	20.6
	Rain2	1.74e-03 ± 3.02e-04	14.7	0.000***	
	log2PA	−7.75e-03 ± 7.57e-03	1.02	0.236	
	Eos	−2.21e-04 ± 1.18e-04	3.43	0.039*	
	IgE	−4.67e-03 ± 3.04e-03	2.28	0.085.	
PA	Intercept	0.467 ± 0.677	0.48	0.455	17.4
	Rain2	1.35e-02 ± 4.35e-03	9.66	0.001**	
	Age	−1.92e-04 ± 2.02e-04	0.90	0.268	
	GIP	−1.03e-04 ± 1.55e-04	0.45	0.478	
	Eos	4.00e-03 ± 2.12e-03	3.64	0.034*	
	Rain*Eos	−3.57e-05 ± 1.19e-05	9.01	0.002**	
Ectosqrt	Intercept	−0.064 ± 0.290	0.05	1.000	2.47
	Rain2	−8.56e-04 ± 3.58e-04	5.70	0.010**	
	Age	2.82e-04 ± 9.64e-05	8.57	0.002**	
	GIsqrt	1.383-02 ± 5.39e-03	6.42	0.006**	
	GIsqrt*Age	−4.58e-06 ± 1.88e-06	5.93	0.009**	
logEos	Intercept	0.751 ± 0.032	549	0.000***	0.07
	Rain2	8.21e-04 ± 1.28e-04	41.0	0.000***	
	GIP	−1.57e-05 ± 8.09e-06	3.77	0.031*	
	Ecto	−3.13e-03 ± 3.15e-03	0.99	0.245	
	Monos	9.39e-05 ± 3.67e-05	6.54	0.006**	
logMonos	Intercept	0.910 ± 0.034	699	0.000***	0.23
	Rain2	4.41e-04 ± 1.57e-04	7.84	0.003**	
	Eos	2.19e-04 ± 8.93e-05	5.99	0.008**	
	IgE	−2.62e-03 ± 1.11e-03	5.56	0.012*	
	IgG	−9.75e-03 ± 3.89e-03	6.27	0.007**	
	Rain*Eos	−7.65e-07 ± 4.40e-07	3.03	0.050.	
logIgE	Intercept	3.97 ± 0.050	6381	0.000***	2.73
	Rain2	3.93e-04 ± 2.08e-04	3.57	0.036*	
	GIP	−1.32e-05 ± 1.51e-05	0.76	0.3126	
	Ecto	−6.76e-03 ± 6.23e-03	1.18	0.2046	
IgGsqrt	Intercept	2.44 ± 0.181	184	0.000***	0.73
	Rain2	8.55e-04 ± 4.51e-04	3.60	0.035*	
	Age	8.66e-05 ± 4.65e-05	3.47	0.038*	
	log2PA	2.26e-02 ± 1.55e-02	2.12	0.095.	
	Ecto	−1.96e-02 ± 1.36e-02	2.08	0.098.	
	Eos	−2.82e-04 ± 2.14e-04	1.73	0.127	
	Monos	−4.57e-04 ± 1.67e-04	7.58	0.003**	

Note.—. *p* ≤ 0.1; *p ≤ 0.05; **p ≤ 0.01; ***p ≤ 0.001.

GIsqrt = transformed GI parasite count (eggs per gram of feces); PA = presence or absence of an anti-PA antibody titer; Ectosqrt = transformed ectoparasite count; logEos = transformed eosinophil count (cells/μl blood); logMonos = transformed monocyte count (cells/μl blood); logIgE = transformed IgE antibody titer (mg/ml serum); IgGsqrt = transformed IgGb antibody titer (mg/ml serum); Rain2 = amount of rainfall (mm) experienced by an individual in the 60 days prior to sampling; log2PA = log_2_ of the dilution used to determine the anti-PA antibody titer.

**Figure 2:**
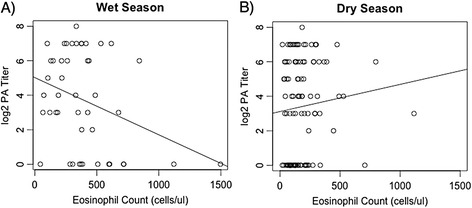
**Interactions between anti-anthrax antibody titers, eosinophil counts, and season. A)** Illustrates a significantly negative relationship between eosinophils and anti-PA titer in the wet seasons, similar to the relationships observed in the PA GEE model. **B)** Shows a positive relationship between eosinophils and anti-PA titer in the dry season, albeit with a less steep slope than in **A**. Plot **B** indicates that the significantly positive relationship between anti-PA titer and eosinophil count observed in the PA GEE model only holds for the dry season.

In the best fitting PA model, as rainfall increased, positive titer prevalence increased, and as eosinophil count increased, titer prevalence increased (Table 2). The interaction term of rainfall with eosinophil count, however, was more highly statistically significant and negative, indicating that animals experiencing higher eosinophil counts were less likely to mount an anti-anthrax immune response than were animals with lower eosinophil counts during the wet (*i.e.* anthrax) season. Figure 2 illustrates that this negative relationship between eosinophils and anti-PA titer is strongly significant in the wet season (Figure 2A), whereas the significantly positive relationship between anti-PA titer and eosinophil count observed in the PA GEE model only holds for the dry season and at a less steep slope than that for the wet season relationships (Figure 2B).

**Figure 3:**
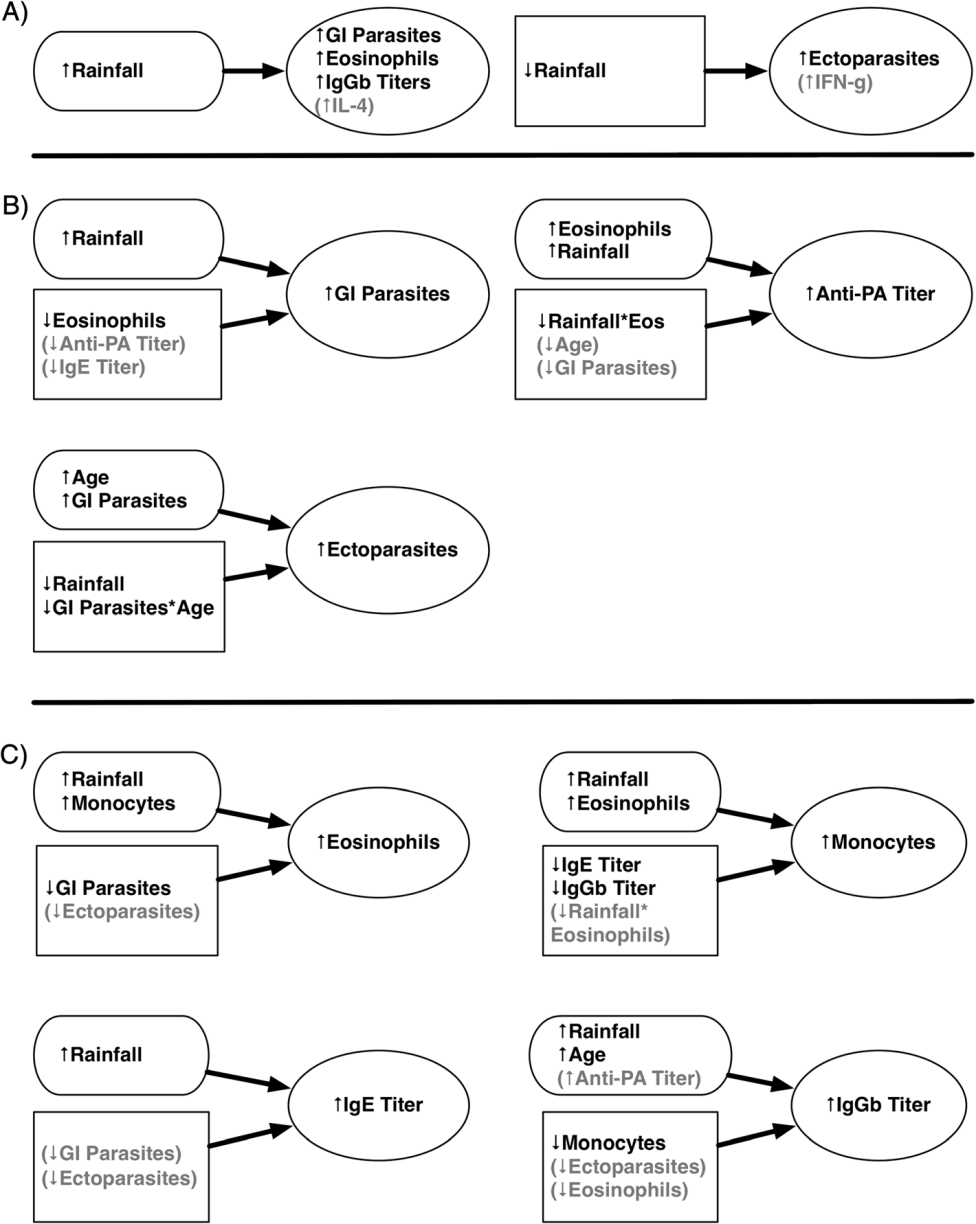
**Illustrated relationships A) between rainfall and other variables for pairwise seasonal comparisons, B) for pathogen GEE models, and C) for immunity GEE models.** Response variables are contained in ovals. Arrows indicate the direction of prediction (e.g. increased rainfall predicted increased GI parasite infection intensities in the GIP GEE model), and do not imply causation. Explanatory variables showing a positive relationship to response variables are in pill-shaped boxes, while those showing a negative relationship to response variables are in rectangular boxes. Significant explanatory or response variables are in black print, while those variables that were included in the models but were statistically not significant are in gray.

In the best fitting ectoparasite model (Table 2), decreased rainfall significantly predicted higher ectoparasite counts, and having more GI parasites significantly predicted higher ectoparasite loads. Relationships for all three pathogen models are illustrated in Figure 3B.

### Immune interaction models

Prior rainfall affected all immunity measures, with increased rainfall associated with increased eosinophils, monocytes, IgE, and IgGb titers (Figure 3C). GI parasite counts were significantly, negatively associated with eosinophil counts, as was observed in the GI parasite GEE model (Table 2). While GI parasite counts and ectoparasite counts were not statistically significant in the IgE model, they both showed a potentially negative association with IgE titer. IFN-γ was significantly negatively associated with animals that had both high eosinophil counts and GI parasite infection intensities (p = 0.000), and with animals that had both a high IgE titer and increased GI parasite infection intensities (p = 0.001). Rainfall and eosinophil counts both significantly positively predicted monocyte counts; however, the interaction between rainfall and eosinophils, was significantly negatively associated with monocyte counts, indicating a complex interplay between these three variables. IgE and IgGb antibody titers both significantly negatively predicted monocyte counts, and monocytes were significantly, negatively correlated with IgGb titers in the IgGb model. Relationships for immunity models are illustrated in Figure 3C.

## Discussion

This study evaluated seasonality of infectious diseases and the seasonal interactions between concurrent infections and immunity in a natural, wet season anthrax-GI parasite system. We found evidence that GI helminth infection intensities, eosinophil counts, IgE and IgGb antibody titers, and possibly IL-4 cytokine concentrations were increased in wet seasons. Ectoparasite infestations and possibly IFN-γ cytokine concentrations were increased in dry seasons, and monocytes and anti-anthrax antibody titers were both negatively associated with interactions between rainfall and eosinophils. These patterns support the hypothesis that zebra experience Th2-type increases in the wet season, that a Th1-type of immunity may predominate in the dry season, and that Th1-Th2 trade-offs may exist in a natural system. We found evidence that this wet season Th2-type immune increase is correlated primarily with GI parasite infections, which may affect anthrax infection dynamics at this time. We have therefore found preliminary evidence that one seasonally bound pathogen may be able to enforce seasonality on another pathogen through host immunomodulation.

### Seasonal immune resource allocation

The seasonal patterns in these immune parameters may partly reflect seasonal nutritional differences. Higher HCT in the wet season likely reflects a higher nutritional state, especially in light of the fact that dehydration often results in an elevated HCT [40]; the fact that HCT in the wet season was still significantly higher than that of the more dehydrated animals in the dry season makes it likely that overall red blood cell numbers were even higher during periods of more rainfall. Higher WBC in the wet season may also reflect this higher plane of nutrition, as may the higher total numbers of eosinophils and monocytes, and higher antibody titers. However, several of these immune parameters had complex relationships with rainfall, pathogen markers, and other immune factors, and thus simple seasonality and nutritional effects cannot completely account for these patterns.

Several of the parasite and immune factors provide evidence that there may exist Th1-Th2 tradeoffs in zebra hosts. Demonstrating such trade-offs in a natural system is powerful in that it helps to ground truth and support similar work previously conducted in controlled laboratory settings. Our work in this natural system also allowed us to examine whether these trade-offs in nature can act in a temporal dimension, as we measured differential immune resource allocation in the wet versus the dry seasons.

As we expected from previous work [22], GI parasite infection intensities were significantly higher in wetter seasons (Figure 1A), likely due to increased survival of environmental life stages with increased moisture [49] and adult worms delaying egg production until it is environmentally advantageous to produce eggs [50]. Eosinophil counts were also significantly higher in the wetter seasons than in the drier ones, consistent with hosts, at a population level, fighting against new GI parasite infections during wetter conditions [51-53] (Figure 1D). Other work in ENP has found that zebra shed significantly more strongyle larvae during wetter seasons than in drier ones, lending further evidence of an overall, population-wide active immune response against new infections during the wet season [W.C. Turner, unpublished data]. Individually, we found a negative relationship between GI parasites and eosinophil counts (Figure 3A and B). Previous studies in wildlife have found a similar pattern, with a strongly negative association between GI helminth FECs and eosinophil counts, even when taking into account other host factors [16,52,54]. These findings suggest that individuals that are predisposed toward mounting stronger Th2 responses (*i.e.* higher eosinophil counts) are more resistant to parasite infections, and thus exhibit lower FECs.

While total monocyte counts were significantly correlated with increased rainfall, they were significantly, negatively correlated with a rainfall-eosinophil interaction effect (Table 2). This perhaps indicates that, while nutritional effects of the wet season may have increased host allocation to immune resources including monocytes, a trade-off between Th2-type eosinophil responses and Th1-type monocyte counts may be occurring. The fact that monocytes were significantly, negatively correlated with the Th2-type immune factors IgE and IgGb titers further supports the hypothesis of a trade-off between these different arms of immunity.

Ectoparasite counts were significantly higher in the drier seasons than in the wetter ones in paired samples but not in unique animal comparisons (Figure 1; Additional file 1: Table B1); this provides evidence that individual susceptibility, likely mediated by immune responses and/or immunomodulation, was at least partly responsible for seasonal differences in ectoparasite infection intensities. Previous studies have found that hard-bodied ticks in most systems prefer higher humidity and intermediate temperatures and are at their lowest numbers on hosts in very hot, dry seasons [55]. As ticks were actually less prevalent on ENP zebras during what should have been their preferred season, this lends more evidence that immunomodulatory conditions influenced tick infestation of hosts.

IgGb antibody titers were significantly greater in wetter seasons, but only for resampled animals, indicating that there are individual differences in immune allocation, immunomodulation, or both (Figure 1; Table 1). While IgGb is likely a Th2-type antibody subtype in equids [45], it is also the most prevalent antibody isotype in equine serum and is potentially involved in protection against other types of pathogens as well [44]. Thus, while changes in IgGb titers may in part reflect seasonal changes in GI parasite infection intensity in ENP zebras, they may also be affected by other factors such as seasonal changes in host nutrition or reproduction.

IL-4 is an instrumental component in protection against or the clearing of many helminth infections, with levels peaking soon following new parasite infections and falling a couple of months after infection attenuation [5,11,56]. While we had a small sample size and non-significant seasonal comparisons, we detected a 15-fold increase in mean IL-4 signal in resampled animals while these animals were experiencing their highest GI parasite infection intensities, consistent with previous findings [5,56]. Similarly, we observed drops in mean IL-4 seen in the dry season as GI infection intensities waned. IFN-γ significantly decreases in the face of increased GI helminth infection intensity and increase after anthelminthic treatment [16]. Though our sample size was small and seasonal comparisons were nonsignificant, our approximately twofold decrease in mean IFN-γ signal in wet seasons when hosts were experiencing peak new GI helminth infections compared to in the relatively parasite-depleted dry season supports previous findings [15,16]. In addition, the significantly negative association between IFN-γ signal and animals with high GI parasite infections in conjunction with either high eosinophil counts or IgE titers lends support for opposing patterns between this Th1-type cytokine and parasites and parasite related immune factors, as would be expected in a parasite driven Th2-type immune skewing.

Thus, the wet season eosinophil peak, combined with significantly higher IgGb antibody titers and preliminary evidence for increased IL-4 and decreased IFN-γ signaling at the same time provides evidence that host immune resources may be skewed toward a Th2-type response in the wet season. The addition of significant inverse relationships between monocytes and IgGb and IgE antibody titers, and between monocytes and eosinophils during times of higher rain, support this hypothesis. The timing of these immune increases in conjunction with the timing of GI helminths infections that are known to affect immunity in a Th2 positive manner suggests that these immune patterns are related to wet season new GI helminth infections. The significant drop in eosinophil counts and IgGb titers in the dry season, coupled with preliminary evidence of increased IFN-γ signaling at that time supports the hypothesis that immunomodulation toward a Th2 response by GI parasites may be attenuated when free-living GI parasite stages are less capable of survival and infecting new hosts.

### Correlates of coinfection with immunomodulatory effects

Previous work in ENP has demonstrated that ungulates ingest significantly more soil in the wet season, likely leading to increased exposure to *B. anthracis* at that time [27]. In support of this, we found that anti-PA titer prevalence was significantly, positively related to rainfall in the PA GEE (Figure 3B), likely reflecting this increased exposure to *B. anthracis* during the wet season, with a subsequent immune response if hosts are exposed to sublethal doses [32]. As we previously found evidence that mean time for negative seroconversion in titer-positive animals is less than six months [32], we are confident that the rainfall-correlated immune signatures represent recent, seasonal *B. anthracis* exposure. This immune signature, coupled with the simultaneously significantly increased rates anthrax deaths supports the assertion that differential exposure to anthrax at least partly drives its seasonality in this system.

However, the wet season increase in Th2-type immune resources observed in our system, in correlation with the timing of peak anthrax incidence, suggests that hosts may also experience seasonally increased anthrax susceptibility shortly following periods of peak GI helminth infection intensity due, at least in part, to immunomodulatory effects and immune tradeoffs. Th2-type skewing by helminths has been found to have significant consequences for hosts fighting against concurrent microparasitic infections [6,57]. In support of this assertion, we also found indirect evidence (through eosinophils) that decreased anti-PA titers were correlated with increased GI parasite infection intensities, and vice versa (Figure 3B). Thus, as GI parasite numbers increase, hosts may be less likely to mount a humoral immune response against anthrax. The significant, negative relationship between the rainfall/eosinophils interaction term and anti-PA titer prevalence in the PA GEE model further supports the hypothesis that this anti-anthrax immune attenuation may be related to GI parasite immunomodulation even in the face of increased anthrax exposure. These anthrax-Th2 immunity relationships held only for the wet season when GI parasite infection intensities were highest (Figure 2A). Similar directional tradeoffs were observed in studies of concurrent GI helminth and tuberculosis infections in buffalo [16,43].

The lack of a stronger, directly negative relationship between GI parasite infection intensity and anti-PA titer magnitude may be due to the ability of animals that ingest low levels of *B. anthracis* in the wet season to mount at least a minor Th1-type immune response, even in the face of strong Th2-skewing. Potian *et al.* [58] found that mice coinfected with GI helminths and lung tuberculosis bacteria were able to mount both Th2 and Th1 responses, respectively, to these pathogens, but only once the parasites colonized separate organs. As animals ingesting very high doses of *B. anthracis* spores are more likely to develop subacute fulminant infection and die [26], animals that have measurable anti-anthrax titers are even more likely to be individuals who experienced low infectious doses while mounting a measureable Th1 response against an established Th2-type skewing. Animals that experience high GI parasite infections and large anthrax doses are also likely at much greater risk for mortality, thus effectively removing very helminth-susceptible and coinfected animals from this study (similar to [43]) but still benefiting anthrax transmission [33]. As we were not able to sample anthrax carcasses for GI parasite and immune parameters, our results may be biased against accurately measuring those animals that succumbed to anthrax due to macroparasite Th2-type skewing.

While studies suggest that ectoparasites are more active in parasitizing hosts during milder and more humid times, ectoparasite infection intensities in our study were both higher on hosts in the dry season in rain group comparisons and were significantly, negatively associated with rainfall in GEE models (Figure 3). Interestingly, higher GI parasite infection intensities were significantly associated with higher ectoparasite counts, despite the opposite interaction of ectoparasites with rainfall (Figure 3). Several studies suggest that a Th1-type immune response is most effective against tick infestations and that ticks can suppress this response in favor of a less effective Th2-type skewing [59-61]. However, our study suggests that a Th2-type immune response is actually more effective in fighting against tick infestations, the more so because otherwise the combination of preferred environmental conditions coupled with a strong Th2 skewing would be expected to result in very high tick burdens in the wet season. As hard-bodied ticks are more capable of survival during dry seasons than are desiccation-prone GI helminth free-living stages [62], the increase in tick infestations in the dry season may represent ticks taking advantage of a decrease in GI parasite-driven Th2 immune pressure at this time. The directionality of these interactions, with GI parasites potentially driving a Th2-type immune response and indirectly affecting ectoparasites, is supported by the fact that rare major histocompatibility complex (MHC) alleles were found to be associated with increased GI parasite infection intensities, and common alleles with increased ectoparasites in ENP zebras [63]. This suggests that GI parasites exert stronger selective pressure at the gene locus in question than do ectoparasites, with GI parasites having higher fitness costs to hosts and likely driving host immune allocation in this system.

## Conclusions

In summary, we measured a complex, interacting set of host-parasite variables that, taken together, support the hypothesis that ENP zebra allocate more immune resources toward a Th2-type of immune response in the wet season, while a Th1-type immunity may prevail in the drier seasons. This Th2-type allocation is likely primarily driven by GI parasite infections, which show strong seasonal fluctuations primarily constrained by external environmental effects on free-living parasite stages. This study is, to our knowledge, one of the first to examine seasonal Th1-Th2 trade-offs in a natural system.

Increased eosinophil counts during wet seasons likely represent an active, and partly successful, immune response against primarily new GI parasite infections. As the interaction between rainfall and eosinophil counts is significantly, negatively correlated with anti-anthrax adaptive immune responses, this may provide evidence for the immunomodulatory effects of GI parasite infections on anthrax susceptibility. As zebra hosts experience significantly increased GI helminth infection intensities and evidence for Th2-type immune resource allocation shortly prior to the population's annual anthrax outbreaks, this suggests that anti-parasite Th2 responses may make hosts less capable of mounting effective Th1-type immune responses against anthrax infections at this time. This increase in susceptibility, combined with evidence of increased *B. anthracis* exposure (increased anti-PA titer prevalence) during the wet season, could be a driver of anthrax outbreaks in this system. Thus, our findings suggest that, through immunomodulation, seasonally constrained pathogens could help force seasonality on other pathogens that are, on their own, not as temporally driven. In addition, by examining the interaction between two environmentally transmitted pathogens, our findings also suggest that certain pathogens can bridge the gap between external and internal ecosystems; through immunomodulatory effects, GI parasites in this system may influence the external ecosystem by altering *Bacillus anthracis* dynamics. While our study is correlative in nature and therefore speculative, this work represents an important first step in understanding the seasonal interplay between macro- and microparasites in a natural system and will hopefully lead to further explorations of these relationships. These findings have important implications both for understanding and predicting anthrax outbreaks, and for understanding the increasingly complex ways in which pathogens can influence hosts, each other, and greater ecosystems.

## Methods

### Study area and species

**Figure 4:**
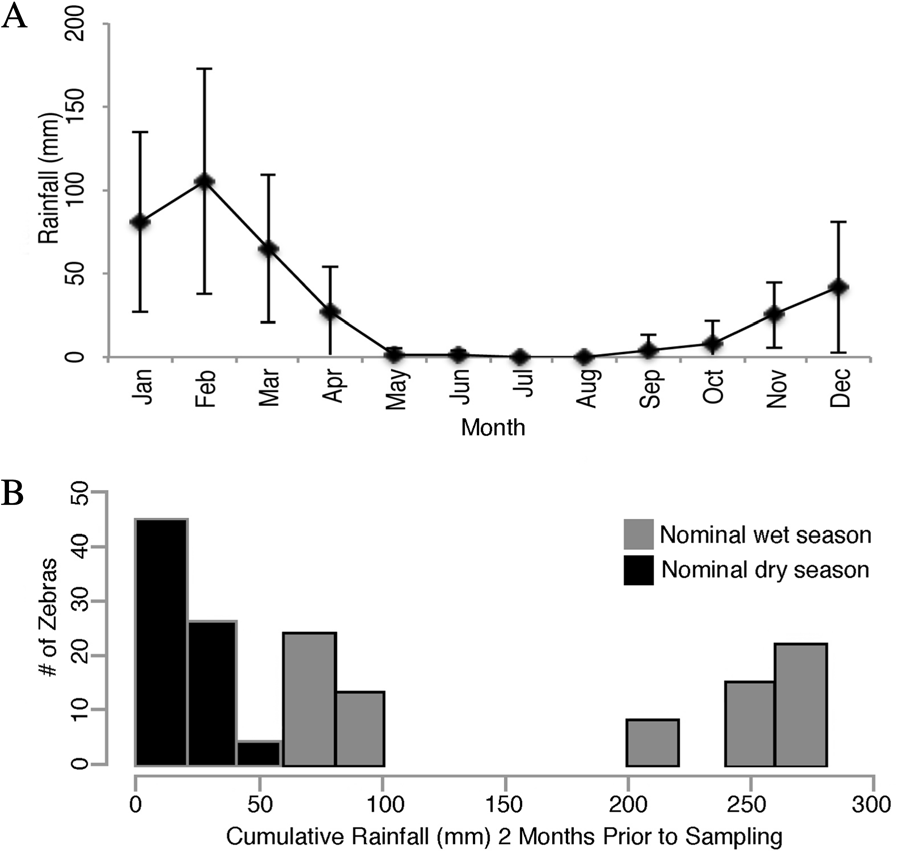
**Monthly rainfall patterns and zebra rainfall experience in sampling seasons. A**. Mean (±SE) monthly Okaukuejo rainfall from 1974–2010. This encompasses the start of the most reliable anthrax sampling in ENP through the years of the current project (2008–2010). **B**. Cumulative rainfall 2 months prior to each zebra capture (Rain2), for all captures over all seasons. The total rainfall in the 60 days prior to capture was determined for each individual zebra capture event, and that number was assigned to that individual-capture as its associated rainfall amount. While we sampled animals in nominally "wet" or "dry" seasons, we saw a clear bimodal pattern in rainfall amounts that did not necessarily align with seasons. This is particularly noticeable in gray bars: Rain2 experienced by animals sampled in the nominal wet season. We therefore used rainfall amounts to assign each individual-capture to a rain season: “wet season,” the high rainfall group, containing individuals that had experienced ≥200 mm rainfall two months prior to sampling; and “dry season,” the low rainfall group, containing individual samplings connected with ≤100 mm rainfall in the two months prior. Black bars: Rain2 experienced by animals sampled in the nominal dry season.

Etosha National Park (ENP) is a 22,915 km^2^ fenced conservation area in northern Namibia, located between 18°30’S-19°30’S and 14°15’E-17°10’E (Additional file 1: Figure A1). Rainfall in ENP is highly seasonal: the rainy season lasts from November through April, with the greatest rainfall occurring during January and February [21,64] (Figure 4). The only perennial water available to the park’s wildlife is found in man-made boreholes, or in natural artesian or contact springs [64].

Plains zebra are one of the most abundant ungulate species in ENP, with a population of approximately 13,000 (95% CI rounded to nearest 100: 10,900-15,000) [Etosha Ecological Institute, EEI, unpublished data 2005].

### Study design and animal capture

We first immobilized and sampled zebra on the plains within approximately 20 km of Okaukuejo, the area in which the majority of anthrax deaths are observed (Additional file 1: Figure A1). In subsequent seasons, zebras were sampled around Okaukuejo and 100 km to the east near Halali following their migration patterns. All animals were safely handled under animal handling protocol AUP R217-0509B (University of California, Berkeley). We originally sampled 45 animals in the wet season, intending to recapture them as many times as possible in five subsequent sampling seasons roughly six months apart (Additional file 1: Table A1) to both control for individual variation and to examine individual versus population level effects of seasonality on immunity and disease.

We obtained whole blood, serum, fecal, and ectoparasite samples from zebra over five seasons between 2008 and 2010 (Additional file 1: Table A2). We conducted a total of 154 zebra capture events, representing 69 unique individuals, with 20 sampled twice, 11 sampled three times, 12 sampled four times, and two sampled five times. We sampled nearly all females to control for sex effects. We sampled only adult animals, further determining age to half a year by combining tooth eruption observations, caliper measurements of upper incisors, and patterns of wear [65,66].

We collected blood from peripheral veins for blood smears, and removed serum for antibody analyses. From blood smears we obtained eosinophil and monocyte counts (Additional file 1: Appendix A). From serum we obtained IgE and IgGb antibody titers, and anti-anthrax (anti-PA) antibody titers using enzyme-linked immunosorbent assays (ELISAs) (Additional file 1: Appendix A). We used an ELISA procedure with wildtype *Bacillus anthracis* PA as coating antigen to measure host anti-PA titers ([32]; Additional file 1: Appendix A). We stimulated whole blood using established protocols, and used these samples to measure IL-4 and IFN-γ cytokine RNA in T cells using RT-PCR (Additional file 1: Appendix A).

We collected feces by observing an individual prior to capture and collecting a homogenized sub-sample within ten minutes of defecation. For animals that were not observed defecating, we collected fecal samples when possible by inserting a gloved hand into the rectum. As capture events took place between 9:00 and 14:00, fecal samples were collected within this same time window, thereby controlling for potential differences in timing of fecal egg shedding [67].

We collected and counted all visible ectoparasites, regardless of life stage, on zebras during capture events. The vast majority of ticks observed were *R. evertsi mimeticus*, a tick species found throughout Namibia in wild equids and greater kudu [68]. These ticks parasitize hosts year-round, with more adults present from November to May and immature stages peaking from February to March and May to September [62].

### Rainfall quantification

The original study design involved sampling based on calendar months of the typical wet/dry seasons. However, annual rainfall variation during the study period caused some individuals sampled during the nominal “wet season” months to be captured during dry conditions. In addition, previous studies in this system determined that gastrointestinal helminth infection intensity in zebras is significantly related to rainfall experienced over the two months prior to sampling [69]. Thus, we determined cumulative rainfall by adding up daily rainfall amounts (in mm) over the 60 days prior to each individual capture event, using local rainfall gauge data. Histograms of cumulative rainfall prior to each individual sampling event revealed a strongly bimodal distribution in the individually-linked rainfall, regardless of calendar-based seasons (Figure 4); we thus chose to group by quantitative rainfall groups. This made both ecological and biological sense: those animals sampled very late in the S3 “wet” season fell into the lower rainfall group as the dry season had begun (Additional file 1: Table A2); and, external moisture greatly influences the within-host development and egg-producing activity of gastrointestinal parasites [70,71]. The rain group seasons were thus: “wet season,” the high rainfall group (individuals that had experienced ≥200 mm rainfall two months prior to sampling); and “dry season,” the low rainfall group (experienced ≤100 mm rainfall).

### Gastrointestinal parasite species and quantification

The gastrointestinal nematodes examined in this host species were in the order Rhabditia, suborder Strongylida, primarily within the superfamily Strongyloidea, and family Strongylidae; this group contains both the “large strongyles” (spp. in the subfamily Strongylinae) and the “small strongyles” (spp. in the subfamily Cyathstominae) [72]. These parasites are oviparous and exhibit a direct life cycle with three, free-living larval stages. The first two molts to the infectious L3 stage occur over one to two weeks, and are highly susceptible to desiccation [23,73]. L3 larvae also require a film of moisture to move [24]; thus, it is unsurprising that previous studies in ENP found a strongly seasonal pattern in zebra strongyle infection intensities, with hosts exhibiting greater new infections during the wet season than in the dry [22]. We evaluated fecal samples for strongyle eggs using a modified McMaster technique for fecal egg counts [74], a commonly used non-invasive method for quantifying parasitism [75] (Additional file 1: Appendix A).

### Statistical analyses

#### Multiple imputation of missing data

Imputation is a method of replacing missing observations with plausible estimates based on available data. Multiple imputation (MI) methods are particularly useful for imputing multivariate missing data, and use available data from multiple predictors and covariates to create a set of datasets for each missing value [76,77]. This method, unlike single imputation methods, provides a variance of an estimate and also estimates the contribution of uncertainty given that the value in question was imputed rather than observed [76,78,79]. While multiple imputation is common in public health research, it has rarely thus far been used in ecological studies.

We imputed missing values for a sample parameter for an individual capture event but did not impute completely missing capture events for an individual animal. We imputed five missing values for IgGb titer (3% of total IgGb data), 15 (10%) for ectoparasite count, and 35 (23%) for GI parasite count. Few of these missing variables were overlapping for the same individual-capture; thus, discarding these cases in favor of complete case analysis would have resulted in disregarding upwards of 79% of the capture events in our analyses. While we used the IFN-γ and IL-4 PCR data in our predictor matrix, we did not impute missing values for these variables given the large number of missing data for each (102 for each of IFN-γ and IL-4).

For imputation, we used the Multiple Imputation by Chained Equations (MICE) method with the 'mice' package [77] in R v2.15.2 [80]. We performed 50 imputation cycles and generated 20 imputations. We averaged the 20 estimates for each data point to produce single mean estimates for use in *t* tests, while we used the multiply imputed datasets directly in our generalized estimating equations and then determined mean estimates for the model parameters [81]. We adjusted standard errors, Wald statistics, and *p* values according to Rubin's rules [82]. As an internal check on the validity of our data imputations, we did the same transformations and group comparison tests on our non-imputed data for each variable; we found that the presence/absence of significant differences between seasons and the directionality of any differences were the same for non-imputed and imputed variables, both before and after correcting for familywise error rates (data not shown). See Additional file 1: Appendix A for a further discussion of multiple imputation and methods used.

#### Seasonal comparisons

We examined the effects of rainfall for our various pathogen and immune factors, first by comparing data from only the first sampling events for animals between the two season types. This ensured that we were only comparing unique individuals, and therefore population-level effects, between seasons, without the possibility of autocorrelation and the issue of repeated measures (Additional file 1: Table A1 A). Due to the fact that we required a comparison of first captures in a wet season with first captures in a dry season, this analysis ultimately used data from only the first wet season of captures (Additional file 1: Table A1 A). We transformed variables to better approximate assumptions of normality when necessary and possible (Additional file 1: Table A3). We compared normalized eosinophil counts, monocyte counts, IgE titers, IgGb titers, and GI parasite counts between seasons using Welch's two-tailed *t* tests. We compared ectoparasite counts and log_2_ anti-PA titers between seasons using two-sided Wilcoxon rank sum tests. We adjusted *p* values to control for the family-wise error rate by using the Holm-Bonferroni method [83]. We then did similar pairwise comparisons across the same animals, between seasons, for first and second captures to examine individual level effects between seasons given individual-level variation that otherwise might have obscured patterns at the population level. We required for this analysis animals that had first been captured in a wet or dry season and subsequently resampled in the opposite season; this ultimately worked out to allow us to use animals from only one wet season for these comparisons (Additional file 1: Table A1 B).

We also compared concentrations of IFN-γ and IL-4 PCR between seasons using two-sided Wilcoxon rank sum tests. Because these tests were only conducted for two seasons’ of samples, grouping them into our regular seasons resulted in a very small sample size for the wet season. We thus modified seasons for these variables only, so that “wet season” included individuals linked with ≥75 mm rainfall and “dry season” included individuals linked with <75 mm rainfall in the two months prior to sampling. This rubric still separated the animals sampled in the last week of April and the beginning of May (the start of the nominal dry season) from those sampled slightly earlier in April. We compared first captures for animals in each season; due to the small sample size for wet season animals in this analysis (*N* = 6), we also compared between seasons for all samples regardless of potential autocorrelation issues. We further compared resampled animals for IFN-γ and IL-4 in their modified seasons using two-sided paired Wilcoxon signed-rank tests.

#### Coinfections and immune relationships

We developed generalized estimating equation (GEE) models using R v2.15.2 and the 'geepack' package [80,84] to examine the correlations between pathogen types and immune parameters. In all models we used a working correlation matrix with a first-order autoregressive relationship because, while individual immune and disease factors are likely correlated through time, these correlations should decrease between later time points and earlier samplings [85].

We developed each GEE using a backwards, stepwise refinement method based on comparing the quasi-likelihood under the independence model criterion (QIC) values between maximal models and models with variables removed [86]. The QIC is analogous to Akaike's information criterion (AIC) for GEEs, which are not strictly likelihood based. After stepwise selection of the main terms, we added interaction terms between the remaining explanatory variables, and further refined the models through backwards, stepwise selection. We subsequently validated the models using established methods [85].

We first used GEE models to address the relationships among our study's three parasite types, seasonal factors, and host parameters (Additional file 1: Table A4). We allowed each parasite to play the role of response variable in separate models to avoid biasing the directionality of parasite interactions and to allow each parasite outcome to be examined from the standpoint of potential predisposing immune factors toward that infection alone. For these same reasons, we then used each of our immune parameters as the response variable in separate models to allow us to more directly examine the cross-relationships between them and the other immune parameters and parasites (Additional file 1: Table A4). We also explored GEE models using IL-4 and IFN-γ as response variables, though with caution as the sample size for these variables was quite low and encompassed only two seasons.

## Additional file

Additional file 1:
**Contains supplemental information for specific methods, as well as additional results for specific models.**

## Abbreviations

ENP: Etosha National Park Namibia
Th1: T helper type 1
Th2: T helper type 2
IgE: Immunoglobulin E
IgGb: Immunoglobulin G subtype b
PA: Protective antigen
IL-4: Interleukin 4
IFN-γ: Interferon gamma
