# Supplementary material for: Gastrointestinal helminths may affect host susceptibility to anthrax through seasonal immune trade-offs
Source: BMC Ecol. 2014 Nov 12;14:27. doi: 10.1186/s12898-014-0027-3 (PMC4247756; doi:10.1186/s12898-014-0027-3)
Supplement: Additional file 1: — Contains supplemental information for specific methods, as well as additional results for specific models. [file 12898_2014_27_MOESM1_ESM.docx]

**Appendix A: Supplementary Methods**

*Hematocrit, Blood Smears, and Counts with Differentials*

Whole blood for hematocrit (HCT) and eosinophil and monocyte counts was collected into Vacutainers (Becton Dickinson, Franklin Lakes, NJ) containing EDTA anticoagulant. We measured HCT, a measure of percent of red blood cells per volume of blood, within five hours of whole blood collection using heparinized capillary tubes and a micro-hematocrit card style reader (StatSpin, Westwood, MA).

To determine eosinophil and monocyte concentrations, we created thin blood smears on glass slides, fixed them with methanol, and stained them with Diff-Quik (Dade Behring, Deerfield, IL). We performed manual total white blood cell (WBC) counts using a compound microscope; we counted cells in ten fields at 40x magnification and multiplied mean cell count per field by 1600 (magnification^2^) to obtain total WBCs per μl of blood. We did differential counts by determining the percent of each of the most common WBC types (neutrophils, monocytes, lymphocytes, eosinophils) in 200 WBCs counted at 40x and multiplying this by total WBC concentration to obtain numbers of eosinophils or monocytes per μl of blood. All counts were done in duplicate and averaged.

*Antibody Concentrations*

For anti-PA (anti-anthrax) antibody titer determination, we used wildtype *Bacillus anthracis* protective antigen (PA) as coating antigen at a concentration of 0.375μl per well. We made serial, twofold dilutions to the ends of rows in duplicate for all samples and negative controls, starting at a dilution of 1:4 and ending at 1:8192 and ran a duplicate negative control full titration series on each ELISA plate. We used goat-anti-horse IgG-heavy and light chain horseradish peroxidase (HRP) conjugate (Bethyl Laboratories, Montgomery, TX) and added TMB substrate (Kirkegaard & Perry Laboratories; Gaithersburg, MD), stopping the reaction with 2N sulfuric acid. We read well absorbance as optical density (OD) at 450nm on a SpectraMax M2 Microplate Reader using SoftMax Pro software v5.3 (Molecular Devices; Sunnyvale, CA). As we had no known, titrated standards to establish a standard curve, we determined the endpoint titers as the log_2_ of the last sample dilution at which the mean OD for that sample at that dilution was greater than the mean OD for all negative controls at that dilution, buffered by a 95% confidence interval determined by the inter-duplicate error at that dilution, across all samples analyzed.

For IgE analysis, we used an ELISA method developed to detect total serum immunoglobulin isotype E (IgE) in domestic horses (described in [1]). We determined IgE concentration in mg/ml of serum by comparing to a titrated, purified IgE standard of known concentration. This is the first known study examining IgE titers in wild equids.

We used a commercially available sheep and goat-anti-horse IgGb ELISA kit (Bethyl Laboratories) to quantify IgGb in serum. We determined concentration of IgGb in serum in mg/ml by comparing to the standard curve and adjusting for dilution amount.

*IL-4 and IFN-*γ *Cytokine Concentrations*

We performed whole blood, *ex vivo* stimulation to investigate interleukin-4 (IL-4) and interferon-gamma (IFN-γ cytokine production by T cells, using an adapted protocol [2]. *Ex vivo* stimulation by antigens has been shown to produce cytokine patterns reflective of those occurring in vivo [3]. Briefly, we added phytohemagglutinin (PHA) to 0.5ml whole blood to achieve a final concentration of 10μg/ml PHA and incubated samples for 24 hours. We examined these parameters for individuals sampled in seasons 2 and 3 only, due to cost constraints of these analyses.

We extracted RNA from stimulated whole blood samples with TRIzol Reagent using the manufacturer’s protocol (Invitrogen, Carlsbad, CA). We treated 1000ng of RNA for each sample with RQ1 DNase (Promega, Madison, WI). For each sample, we prepared cDNA using SuperScript III reverse transcriptase (RT) (Invitrogen) and a negative control sample lacking RT. cDNA reactions were primed with poly(dT). We performed quantitative PCR using a Step One Plus RT-PCR system (Applied Biosystems, Foster City, CA) with Platinum *Taq* DNA polymerase (Invitrogen) and EvaGreen (Biotium, Hayward, CA). The target genes of interest were domestic equine IFN-γ and IL-4, and we used GAPDH as our housekeeping gene (primer sequences from Ainsworth *et al.* 2003) [4, 5]. Primers were provided by Elim Biotech (Hayward, CA).

For one sample on each plate, we analyzed serial ten fold dilutions for each primer pair in duplicate. Using C_T_s (cycle number at which product is reliably detected) for these dilutions, we constructed a relative standard curve for each target gene to calculate the log_10_cDNA amount as a proxy for concentration (ng) of target RNA in each sample. We normalized these transcript levels to those of GAPDH for each sample by calculating the cytokine:GAPDH ratio.

*Modified McMaster Protocol*

We used a modified McMaster method to count parasite eggs in feces [6]. Briefly, we combined 4g of homogenized fecal matter with 56ml of a saturated NaCl solution (specific gravity 1.2), removed any large debris with a strainer, and obtained a homogenized filtrate. We placed an aliquot of filtrate into each chamber of a McMaster slide and counted the number of eggs observed in each chamber using a compound microscope at 10x magnification. We obtained a measure of eggs per gram of feces by adding the number of eggs for both chambers and multiplying by 50.

Fecal egg counts (FECs) provide an accurate estimate of how the input of parasite eggs into the environment varies with other factors of interest [7]. While the actual relationship between fecal egg count and total nematode burden within a host is of unknown specificity and sensitivity, these counts provide a nonlethal and often noninvasive method for estimating these infection burdens [8–10]. In addition, we previously found that fecal water content had no effect on seasonal and age-related patterns in strongyle egg counts, thus increasing our confidence regarding the overall accuracy of this measurement [11].

*Multiple Imputation of Missing Data*

Multiple imputation is most often used in human public health studies in which some data are missing for individuals sampled repeatedly over time [12–14]. Comparisons of analyses using multiply imputed datasets versus complete case analysis (CCA), in which cases with any missing data are eliminated from the analysis, have found that MI produces much less biased results. This is true when both small and large amounts of data points are missing [13, 15]. In addition, CCA has been found to be appropriate only when data are known to be absolutely missing completely at random (MCAR); because there are often underlying, potentially unobserved causes for missing data, using CCA is often suboptimal [12, 15, 16].

For imputation, we used the Multiple Imputation by Chained Equations (MICE) method with the 'mice' package [17] in R v2.15.2 [18]. This method specifies the imputation model for each variable by building, and iterating over, a set of conditional densities for each variable. We built our predictor matrix by first using all variables in this study [16], and then refined the predictor matrix for each variable to avoid collinearity. We preserved all data transformations by passively imputing each transformed variable linked to its original variable [17]. We validated our imputations by confirming convergence, examining density plots and strip plots to ensure that imputed values overlapped existing data, and comparing distributions of observed versus imputed data based on propensity scores [17].

**Appendix A Figure Captions**

**Figure A1.** **Etosha National Park in northern Namibia.**

The Etosha Ecological Institute is located in Okaukuejo in the center of the park; the majority of animal sampling for this study occurred in the nearby surrounding area, within a radius of approximately 20km (in the plains outside of the salt pans). During drier seasons, some sampling took place up to 100km to the east of Okaukuejo, around the Halali plains, and 15km south of Okaukuejo. The majority of anthrax cases in the park occur within the dotted outline around Okaukuejo.

**
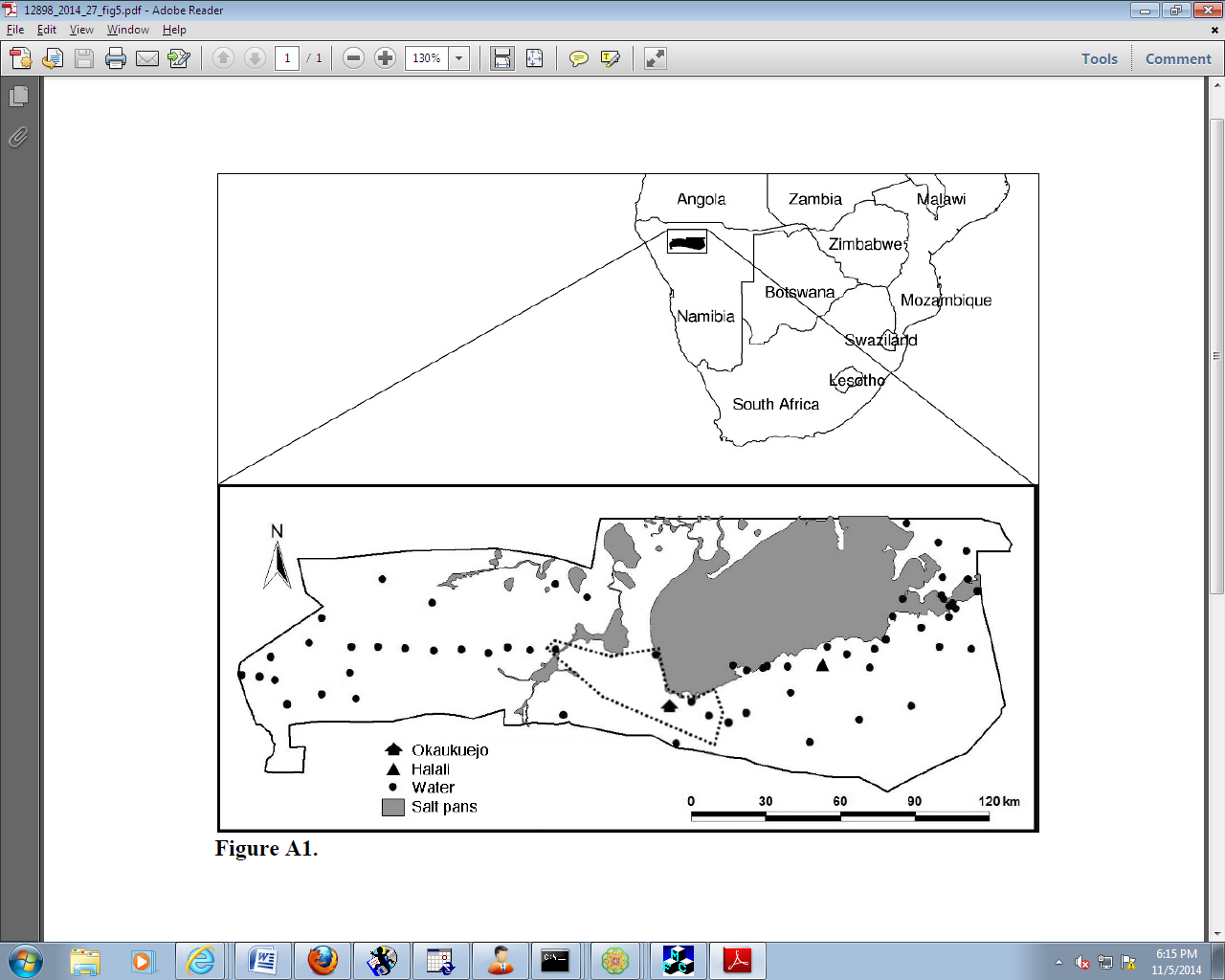
**

**Appendix A Tables**

**Table A1. A. Number of zebra captured in each season for first captures only, grouped by seasons. B. Number of zebra captured in each season for paired recaptures only, grouped by seasons.**

| A. | Capture Season | Cap1Wet | Cap1Dry | B. | Capture Season | Cap1Wet | Cap2Dry |
| --- | --- | --- | --- | --- | --- | --- | --- |
|  | S1 | 45 | 0 |  | S1 | 32 | 0 |
|  | S2 | 0 | 14 |  | S2 | 0 | 23 |
|  | S3 | 0 | 6 |  | S3 | 0 | 8 |
|  | S4 | 0 | 4 |  | S4 | 0 | 1 |
|  | S5 | 0 | 0 |  | S5 | 0 | 0 |

Note.— S1-S5 are the numbered five capture seasons for which S1 and S3 were nominal wet seasons and S2, S4, and S5 were nominal dry seasons; Cap1 = capture 1; Cap2 = capture 2 for the same individual; Wet = wet season (experience of cumulative rainfall >200mm over the two months prior to capture); Dry = dry season (experience of cumulative rainfall <100mm over the two month prior to sampling). There were no resampled animals that fell into Cap1Dry or Cap2Wet groups.

**Table A2. Zebra capture seasons, timing, animals involved, and samples taken.**

| CS | NS | Date  (Mo/Yr) | Blood | Feces | Ticks |
| --- | --- | --- | --- | --- | --- |
| S1 | Wet | 3-4/08 | 45(45,0) | 38(38,0) | 45(45,0) |
| S2 | Dry | 10-11/08 | 36(14,22) | 29(17,12) | 18(0,18) |
| S3 | Wet | 4-5/09 | 35(6, 29) | 32(4,28) | 30(5,25) |
| S4 | Dry | 9-11/09 | 13(4,9) | 10(3,7) | 13(4,9) |
| S5 | Dry | 8/10 | 25(0,25) | 14(0,14) | 19(0,19) |
| Totals |  |  | 154(69,85) | 123(62,61) | 125(54,71) |

Note.— CS = Capture Season; NS = Nominal Season. Data refer to Total#(#New, #Resampled), where New = new individuals and their samples and Resampled = animals resampled at least once in that season and their corresponding samples collected. Ticks = number of zebras sampled for total tick burden.

**Table A3. List of variables used in models, with their abbreviations and descriptions.**

| Variable | Abbreviation | Description |
| --- | --- | --- |
| Cumulative rain 2 months prior | Rain | Rain (mm) experienced by an individual in 60 days prior to a capture event |
| Individual age | Age | Age (years) at a sampling event, determined first by dental wear |
| GI parasite burden | GIP, or GIsqrt when square root transformed | GI parasite infection intensity (nematode eggs/gram of feces) |
| Sublethal anthrax exposure | log2PA or PA | Anti-PA antibody titer as measured in log_2_ of final dilution (log2PA) or as presence or absence of a titer (PA) |
| Ectoparasite burden | Ecto, or Ectosqrt when square root transformed | Total number of ticks |
| Eosinophil count | Eos, or logEos when log_10_ transformed | Number of eosinophils/μl of blood |
| Monocyte count | Monos, or logMonos when log_10_ transformed | Number of monocytes/μl of blood |
| IgE Titer | IgE, or logIgE when log_10_ transformed | Serum concentration of IgE antibodies (μg/ml) |
| IgGb Titer | IgG, or IgGsqrt when square root transformed | Serum concentration of IgGb antibodies (mg/ml) |

**Table A4. Maximal generalized estimating equation models evaluated.**

| Pathogen Models |  |  |  |
| --- | --- | --- | --- |
| GIsqrt | ~ | Rain2 + Age + log2PA + Ecto + Eos + Monos + IgE + IgG | |
| PA | ~ | Rain2 + Age + GIP + Ecto + Eos + Monos + IgE + IgG | |
| Ectosqrt | ~ | Rain2 + Age + GIsqrt + log2PA + Eos + Monos + IgE + IgG | |
|  |  |  | |
| Immune Models |  |  | |
| logEos | ~ | Rain2 + Age + GIP + log2PA + Ecto + Monos + IgE + IgG | |
| logMonos | ~ | Rain2 + Age + GIP + log2PA + Ecto + Eos + IgE + IgG | |
| logIgE | ~ | Rain2 + Age + GIP + log2PA + Ecto + Eos + Monos + IgG | |
| IgGsqrt | ~ | Rain2 + Age + GIP + log2PA + Ecto + Eos + Monos + IgE | |

**References**

1. Wagner B, Radbruch A, Rohwer J, Leibold W: Monoclonal anti-equine IgE antibodies with specificity for different epitopes on the immunoglobulin heavy chain of native IgE. Vet Immunol Immunopathol 2003, 92:45–60.

2. Thurm CW, Halsey JF: Measurement of cytokine production using whole blood. Curr Protoc Immunol 2005, 7.18:B.1–B.12.

3. Heinzel FP, Sadick MD, Mutha SS, Locksley RM: Production of interferon y, interleukin 2, and interleukin 10 by CD4+ lymphocytes in vivo during healing and progressive murine leishmaniasis. Proc Natl Acad Sci U S A 1991, 88:7011–7015.

4. Ainsworth DM, Grunig G, Matychak MB, Young J, Wagner B, Erb HN, Antczak DF: Recurrent airway obstruction (RAO) in horses is characterized by IFN-g and IL-8 production in bronchoalveolar lavage cells. Vet Immunol Immunopathol 2003, 96:83–91.

5. Ainsworth DM, Appleton JA, Eicker SW, Luce R, Flaminio J, Antczak DF: The effect of strenuous exercise on mRNA concentrations of interleukin-12, interferon-gamma and interleukin-4 in equine pulmonary and peripheral blood mononuclear cells. Vet Immunol Immunopathol 2003, 91:61–71.

6. Gibbons LM, Jacobs DE, Fox MT, Hansen J: McMaster egg counting technique. In R Vet Coll Agric Organ United Nations Guid to Vet diagnostic Pathol; 2005.

7. Turner WC, Getz WM: Seasonal and demographic factors influencing gastrointestinal parasitism in ungulates of Etosha National Park. J Wildl Dis 2010, 46:1108–1119.

8. Stear MJ, Bishop SC, Duncan JL, McKellar QA, Murray M: The repeatability of faecal egg counts, peripheral eosinophil counts, and plasma pepsinogen concentrations during deliberate infections with Ostertagia circumcincta. Int J Parasitol 1995, 25:375–380.

9. Wilson K, Bjørnstad ON, Dobson AP, Merler S, Poglayen G, Read AF, Skorping A: Heterogeneities in macroparasite infections: patterns and processes. In Ecol Wildl Dis. Edited by Hudson PJ, Rizzoli A, Grenfell BT, Heesterbeek H, Dobson AP. Oxford, UK: Oxford University Press; 2001:6–44.

10. Seivwright LJ, Redpath SM, Mougeot F, Watt L, Hudson PJ: Faecal egg counts provide a reliable measure of <I>Trichostrongylus tenuis</I> intensities in free-living red grouse <I>Lagopus lagopus scoticus</I>. J Helminthol 2004, 78:69–76.

11. Turner WC, Cizauskas CA, Getz WM: Variation in faecal water content may confound estimates of gastro-intestinal parasite intensity in wild African herbivores. J Helminthol 2009, 84:99–105.

12. Little RJ, Rubin DB: Causal effects in clinical and epidemiological studies via potential outcomes: Concepts and analytical approaches. Annu Rev Public Health 2000, 21:121–145.

13. Van der Heijden GJMG, Donders a RT, Stijnen T, Moons KGM: Imputation of missing values is superior to complete case analysis and the missing-indicator method in multivariable diagnostic research: a clinical example. J Clin Epidemiol 2006, 59:1102–9.

14. Johansen NB, Vistisen D, Brunner EJ, Tabák AG, Shipley MJ, Wilkinson IB, McEniery CM, Roden M, Herder C, Kivimäki M, Witte DR: Determinants of aortic stiffness: 16-year follow-up of the Whitehall II study. PLoS One 2012, 7:e37165.

15. Vergouw D, Heymans MW, van der Windt D a WM, Foster NE, Dunn KM, van der Horst HE, de Vet HCW: Missing data and imputation: a practical illustration in a prognostic study on low back pain. J Manipulative Physiol Ther 2012, 35:464–71.

16. Rubin DB: Multiple imputation after 18+ years. J Am Stat Assoc 1996, 91:473–489.

17. Van Buuren S, Groothuis-Oudshoorn K: mice: Multivariate imputation by chained equations in R. J Stat Softw 2011, 45:1–67.

18. Team R: R Development Core Team. R A Lang Environ Stat Comput 2013.
